# Individualized therapy of HHT driven by network analysis of metabolomic profiles

**DOI:** 10.1186/1752-0509-5-200

**Published:** 2011-12-20

**Authors:** Neema Jamshidi, Franklin J Miller, Jess Mandel, Timothy Evans, Michael D Kuo

**Affiliations:** 1 Department of Radiological Sciences, University of California, Los Angeles, USA; 2 Department of Radiology, University of California, San Diego, USA; 3 Department of Internal Medicine, University of California, San Diego, USA; 4 Department of Medicine, University of California, San Francisco-Fresno, USA

## Abstract

**Background:**

Hereditary Hemorrhagic Telangiectasia (HHT) is an autosomal dominant disease with a varying range of phenotypes involving abnormal vasculature primarily manifested as arteriovenous malformations in various organs, including the nose, brain, liver, and lungs. The varied presentation and involvement of different organ systems makes the choice of potential treatment medications difficult.

**Results:**

A patient with a mixed-clinical presentation and presumed diagnosis of HHT, severe exertional dyspnea, and diffuse pulmonary shunting at the microscopic level presented for treatment. We sought to analyze her metabolomic plasma profile to assist with pharmacologic treatment selection. Fasting serum samples from 5 individuals (4 healthy and 1 with HHT) were metabolomically profiled.

A global metabolic network reconstruction, Recon 1, was used to help guide the choice of medication via analysis of the differential metabolism between the patient and healthy controls using metabolomic data. Flux Balance Analysis highlighted changes in metabolic pathway activity, notably in nitric oxide synthase (NOS), which suggested a potential link between changes in vascular endothelial function and metabolism. This finding supported the use of an already approved medication, bevacizumab (Avastin). Following 2 months of treatment, the patient's metabolic profile shifted, becoming more similar to the control subject profiles, suggesting that the treatment was addressing at least part of the pathophysiological state.

**Conclusions:**

In this 'individualized case study' of personalized medicine, we carry out untargeted metabolomic profiling of a patient and healthy controls. Rather than filtering the data down to a single value, these data are analyzed in the context of a network model of metabolism, in order to simulate the biochemical phenotypic differences between healthy and disease states; the results then guide the therapy. This presents one approach to achieving the goals of individualized medicine through Systems Biology and causal models analysis.

## Background

Complex diseases with multi-factorial etiologies often have multiple alternative pathways leading to a particular pathophysiological state, with a wide range of resulting phenotypes. Such diseases provide a significant diagnostic and treatment challenge, and will require individual-specific, personalized treatments. Hereditary Hemorrhagic Telangiectasia (HHT) is an example of a Mendelian genetic disease with broad variability in presentation and involvement of different organs. Arteriovenous malformations (AVM) can occur in multiple beds, including the brain, liver, lungs, and nose [1]. A challenge for treatment of this disease is selecting an appropriate treatment for a given clinical setting. Since many diseases affect metabolism directly or indirectly, the field of metabolomics has rich potential for biomarker applications. A challenge however is that a metabolomic profile alone may not provide any direction for treatment, since there is not biologically coherent integration of these data. Metabolic network reconstructions can provide the framework for integration and analysis of these data [2-16]. We considered the treatment of an individual patient and through comparative analysis of her metabolomic profile with non-HHT individuals, differences were identified using constraint-based analysis of a global human metabolic network reconstruction, Recon 1 [17].

The patient in this study is a female who began experiencing syncopal episodes at rest and during exertion at 21 years of age. She was found to have a 28 mm atrial septal defect (ASD), for which percutaneous closure was attempted, but failed, ultimately requiring open heart surgery. The ASD repair resolved the syncopal episodes, however her dyspnea continued unabated to the point where she could not walk her dog without getting chest pain and expectorating small amounts of blood streaked sputum. Further investigation with contrast echocardiography showed she had a large right to left shunt at the pulmonary capillary level but had no treatable AVMs on computed tomography (CT) scan of the chest. Her resting oxygen saturations were 97-99% but with effort could decrease to the high 80's. Her pulse was regular but varied between 90 and 120 beats per minute and variations in blood pressure were also noted and treated medically when elevated. Because of a strong family history cancer of the colon and some occasional blood streaks in her stool, a colonic endoscopy was done revealing several small polyps in her small intestine and telengiectasias in her large intestine that were ablated with laser therapy. Her Endoglin and ALK-1 gene analysis were negative including SMAD-4. Based upon these findings as well as a family history of familial adenomatous polyposis and a sibling with similar diffuse AVMs, a presumed diagnosis of HHT was given based on the Curacao Criteria [18].

In this individual case study, untargeted, quantitative plasma metabolomic profiling is carried out in a patient and healthy controls. We sought to use constraint-based modeling of metabolism on an organism scale to identify potential differences in metabolism between the non-HHT and HHT patient through identification of biomarker signature profiles (as opposed to single biomarkers) that can be linked to different functional states. These differences were then used to support the use of particular drug treatments; post-treatment profiling was carried out to assess whether the treatment revised the biochemical pathways accordingly.

## Methods

Fasting plasma samples drawn from 5 healthy individuals (no chronic medical conditions, no current daily medications or herbal supplements; ages 21-37) and 1 patient with HHT (age 24) (UCLA Institutional Review Board retrospective case report exemption was obtained). The blood from the healthy volunteers was taken as part of a general protocol for internal standardization/normalization at UCSD. A set of samples from individuals in the study were used and are currently part of an ongoing study that allows acquisition and analysis of blood from healthy individuals as well as HHT patients (UCLA Institutional Review Board Protocol Number 11-000843-AM-00003). Blood draws from two different time points had been previously obtained from two of the healthy individuals and for the patient, resulting in a total of 9 samples. Additionally one individual provided a fasting as well as non-fasting blood sample, which served as a non-fasting positive control. Written informed consent was obtained from the patient for publication of this report. A copy of the written consent is available for review by the Editor-in-Chief. All of the experimental research with volunteers and the patient were performed with the approval of the ethics committees of the institutions where the studies were done.

Blood samples were obtained by a licensed health care practitioner and spun down for 10 minutes at approximately 3000 rpm at 20 C. The supernatant was stored in a freezer, and after all samples were obtained, were sent for processing and profiling (Chenomx Inc, Edmonton, Alberta). The samples were filtered using 3 kDa molecular weight cut-off filters (Nanosep 3K Omega microcentrifuge filter tubes). Internal standard solution was added to each sample solution, and the resulting mixture was vortexed for 30s. 600 μL of the mixed solution was transferred to an NMR tube for data acquisition. Spectra were acquired on a 600 MHz Varian INOVA spectrometer with 32 scans per sample at 298 K. Spectra were processed and CNX files were generated using the Processor module in Chenomx NMR Suite 6.0. Metabolites were identified and quantified using the Profiler and Library Manager modules in Chenomx NMR Suite 6.0, using a metabolite reference library of 297 metabolites.

### Sample analysis

**Figure 1:**
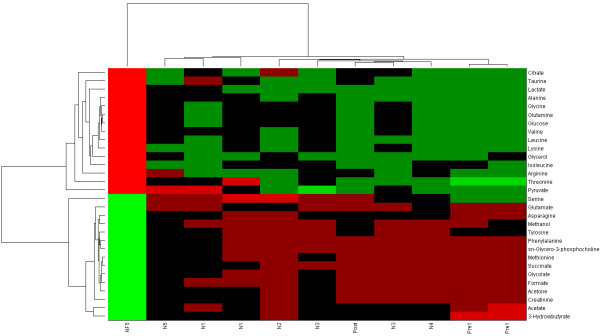
**Two way clustering of plasma metabolomic profiles of non-HHT patients (N1-N5) and HHT patient (Pre1 and Post)**. All of the blood draws were taken when patients were fasting, except for NF5 which was a non-fasting profile for individual N5. The non-fasting profile is clearly distinguished from the other profiles. Also blood draws for the HHT patient prior to bevacizumab treatment (Pre1) is in a distinct branch from the other profiles. Note that the post-treatment profile (Post) for the HHT patient is concordant with the non-HHT individual's profiles.

Unsupervised hierarchical clustering showed clear separation between the healthy individuals and the patient. There were not enough replicates to apply conventional statistical tests (seven measurements in one group and two in another), however two-way hierarchical clustering with unweighted average distance linkage of the metabolite profiles demonstrated a clear separation between the patient and healthy individuals, which also drove the separation in the clustergram (Figure 1). The distance measure in Figure 1 is the infinity norm, but similar separations were observed with other norms (e.g. 1- and 2-norms). Separation of the pre-treatment and post-treatment of the HHT patient as well as re-grouping of the post-treatment HHT patient with the non-HHT individuals was observed with Principal Component Analysis as well (see Additional File 1).

Differences in metabolism between non-HHT individuals and the HHT patient were assessed using the plasma metabolomic profiles. Specific metabolites that were quantitatively and qualitatively different between the non-HHT and HHT patient were determined by identifying those metabolites whose maximum concentration in one group was less than the minimum of the other group or vice versa. This resulted in two sets of metabolites that were different in the two conditions; a high set and a low set. The two general classes of metabolites were labeled the as 'ketone group' and the 'amino acid/carbon rich group' and used to define qualitative metabolic pseudo-reactions.

### Network analysis

Recon 1, a global human metabolic network reconstruction [17], with elementally charge and mass balanced equations can be used for analysis of transcriptomic, proteomic, and metabolomic data [19] using constraint-based analysis methods [20,21].

We predicated our approach on the assumptions that following 8 hour fasting overnight, the body is at or moving towards a homeostatic state, the full content of Recon 1 represents the set of metabolic interactions in the human body, and that changes in plasma profile reflect net changes in uptake and/or secretion of different metabolites with the set of all organs. We briefly describe the constraint-based analysis approach for Flux Balance Analysis, noting there is a rich literature on the subject that the interested reader can pursue in greater detail [9,20,21].

(1) S⋅v=0

In which **S **is the mxn stoichiometric matrix with m metabolites, n reactions, with each column representing a metabolic (or transport) reaction and **v **is a vector of fluxes corresponding to each reaction in the network.

Constraints area applied to the network as upper and lower bounds on the fluxes,

(2) α≤v≤β

In which the **α **vector specifies reaction flux lower bounds and the **β **vector specifies reaction flux upper bounds.

The next step requires specification of an objective function which will then be minimized or maximized. We consider linear objective functions only in this study, thus,

$$\max\left(c^{\text{T}}\cdot v\right)\;\text{or}\;\min\left(c^{\text{T}}\cdot v\right)$$

are the objectives, in which **c **is a signed, binary vector. Flux Variability Analysis (FVA) [22] is a method in which every reaction in a network is maximized and minimized under a specified set of constraints, thus providing a 'bounding box' on the current state. This approach can be useful when one is interested in providing a general characterization of the fluxes in one state and how the extrema change from one condition to another. This method has demonstrated interesting results in the analysis of human metabolism in the general as well as context specific conditions [23,24].

The intravascular space is available for uptake and secretion of metabolites with all organs of the body, thus changes in metabolomic profiles may be interpreted in the context of a global human metabolic network. Direct flux data for particular reactions were not available, so the two qualitatively different profiles, derived from the quantitative differences in the metabolomic profiles of plasma were used to define two sets of different transport constraints. Changes in the simulation conditions provided information about the changes in the metabolite profiles, and were used to define constraints (see Additional File 2). The changes in metabolite profiles reflect differences in metabolic states between the HHT patient and the non-HHT individuals. While the underlying cellular objectives cannot be directly deciphered from these data, the changes in metabolite profiles can be implemented as a constraint to be satisfied, through formulation of a pseudo-reaction, grossly similar to the use of non-growth associated biomass objectives in bacteria [25].

The COBRA toolbox [26] was used to carry out calculations in Matlab (The Mathworks, Natick, MA) with GLPK (GNU software, http://www.gnu.org) for the linear optimization steps, using Recon 1 [17]. The cytochrome oxidase reactions were adjusted to be completely charge and mass balanced, as previously described [5,27] (see Additional File 3 for the stoichiometric matrix). FVA was performed on Recon 1 with a 'rich media' set of uptake constraints (permission to uptake 20 amino acids, glucose, palmitate, oxygen and exchange of protons and water). The flux variability results were used to define upper and lower bounds on the reactions for an 'open set' of uptake constraints.

The set of metabolites that were elevated in the HHT patient (i.e. whose minimum measured concentrations in the HHT patient were greater than the maximum measured concentrations of all of the non-HHTs) largely consisted of ketones and were dubbed the 'ketone group'. Conversely, the set of metabolites that were decreased in the HHT patient (i.e. whose maximum measured concentrations in the HHT patient were less than the minimum measured concentrations of all of the non-HHTs) primarily were amino acids and were dubbed the 'amino acid group'. The production (i.e. exchange) maxima for the 'ketone group' and 'amino acid group' metabolites were used to specify coefficients for two different pseudo-objective reaction constraints, one representing the HHT patient and the other representing the non-HHT profiles. The 'ketone group' objective was scaled by one-half in order to be able to maintain a feasible solution in the null space (the general set of constraints on the model could not simultaneously satisfy the individual ketone group metabolite maxima). These objectives represent the different non-biomass demands for each condition (using the FVA calculations to avoid setting an infeasible coefficient, see Additional File 2, 'metabolites' tab). The amino acid group included tyrosine, taurine, serine, glycine, alanine, citrate, lactate, methanol, and creatinine. The ketone group included acetone, formate, and acetate. After each model (HHT and non-HHT) was optimized for their respective pseudo-objective and fixed at that value, FVA was again carried out. Subsequently the Flux Span (difference between maximum and minimum flux attainable for all reactions in a particular condition) and the Flux Span Ratio (the reaction-wise ratio of the Flux Span between two conditions) were calculated for comparison.

## Results and Discussion

As a means to evaluate possible related metabolic derangements, fasting plasma metabolomic profiling was carried out (see METHODS). Fasting blood samples from 4 non-HHT individuals, was obtained and metabolomic profiling carried out as well. Two of the non-HHT individuals provided multiple fasting samples (on two different occasions) and another individual provided a non-fasting sample. The serum samples were analyzed using NMR spectroscopy with subsequent quantification and identification of 30 metabolites.

**Figure 2:**
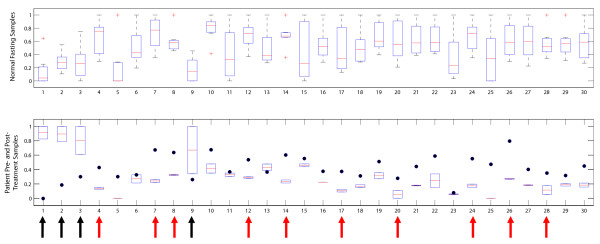
**Metabolites that were relatively increased in non-HHT individuals but decreased in the HHT patient (red arrows) included alanine, glycine, lysine, serine, tyrosine, glutamine, and creatinine**. Metabolites that were relatively increased in the HHT patient (black arrows) included 3-hydroxybutyrate and acetate. Following treatment with bevacizumab, the HHT patient's profile (bottom panel) reversed the trend on the above metabolites, and developed a more 'normal' profile. Metabolite names: 1: 3-Hydroxybutyrate, 2: Acetate, 3: Acetone, 4: Alanine, 5: Arginine, 6: Asparagine, 7: Citrate, 8: Creatinine, 9: Formate, 10: Glucose, 11: Glutamate, 12: Glutamine, 13: Glycerol, 14: Glycine, 15: Glycolate, 16: Isoleucine, 17: Lactate, 18: Leucine, 19: Lysine, 20: Methanol, 21: Methionine, 22: Phenylalanine, 23: Pyruvate, 24: Serine, 25: Succinate, 26: Taurine, 27: Threonine, 28: Tyrosine, 29: Valine, 30: sn-Glycero-3-phosphocholine.

Clustering of the samples demonstrated clear separation of the non-HHT individuals fasting sample, the HTT patient (pre-treatment), and the single non-HHT individual non-fasting sample (Figure 1). The trends in the non-fasting sample were in opposite direction in comparison to all of the other samples, consistent with different feeding states. The HHT patient's two fasting blood samples were in a different branch of the clustergram as well (Figure 1). Since only two measurements of the patient (pre-treatment) were available, it was not possible to carry out t-tests or ANOVA analyses and a different criterion was required to identify the most distinctive qualitative differences in the profiles. Comparisons between the non-HHT and HHT patient (pre-treatment) were carried out in the following manner: metabolites whose minimum value in the non-HHTs was greater than the maximum value in the HHT patient's measurements or whose maximum value in the non-HHTs was less than the minimum value in the HHT patient's were used to define qualitative transport constraints for a non-HHT and HHT network (Figure 2).

A global metabolic human network, Recon 1 [17], was used to analyze the metabolomic data in a biologically germane context [19]. Since the intravascular compartment interacts with all organs of the body as both a 'source' and 'sink' for nutrients and waste, respectively, the plasma metabolite measurements were used as constraints on the global network, to grossly approximate whole organism metabolism. The two different conditions were used to specify constraints, resulting in two different networks. The two different networks were then globally assessed using FVA and compared for differences in individual reactions and pathways.

**Figure 3:**
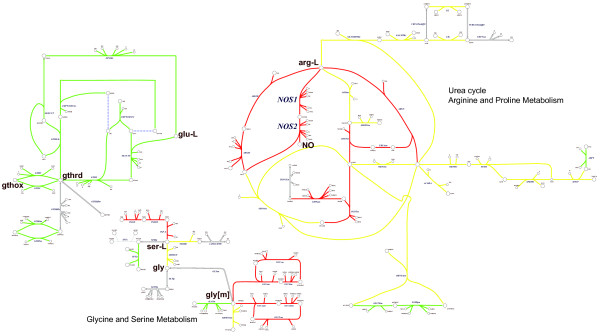
**Reactions and pathways that were found to be different when comparing FVA profiles for the healthy individuals as compared to the HHT patient (prior to bevacizumab treatment).** Green fluxes represent increased flux span ratios and red fluxes represent decreased flux span ratios, for a non-HTT individual compared to an individual with HHT (e.g. red colored reactions indicate pathways with increased flux spans and maximum fluxes in HHT). Yellow shaded reactions demarcate reactions with intermediate changes in flux span ratios. Abbreviations: NOS1/NOS2: Nitric Oxide Synthase, NO: nitric oxide, arg-L: L-arginine, ser-L: L-serine, gly: glycine (in cytosol), gly[m]: glycine (in mitochondria), glu-L: L-glutamate, gthrd: glutathione (reduced), gthox: glutathione (oxidized).

Comparison of the non-HHT and HHT patient networks using flux span ratios (see METHODS) demonstrated decreased energy production in the HHT patient, reflective of a 'starvation-like' state. Interestingly, there were noted increased flux potentials in nitrogen handling and disposition pathways in the HHT patient, notably with nitric oxide synthase (NOS) (Figure 3). This observation brought to light a potential link between vascular endothelial function and the changes in vascularity found in HHT, with connections to metabolism [1,28,29]. There has been evidence suggesting that VEGF can decrease blood pressure through increased nitric oxide production and conversely, that inhibition of VEGF can increase blood pressure, at least in part, through the same mechanism [30-32]. The potential increase in NOS activity based on the network analysis of the metabolic profile supported the use of bevacizumab (Avastin), an anti-VEGF drug. The HHT patient underwent treatment with bevacizumab at a dose of 5 mg/kg every 2 weeks for 6 total infusions, at which point another fasting blood draw was obtained. The patient had a mild response to therapy which lasted for 2 months. Surprisingly, the patient's profile had changed to become more similar to the control individuals. When clustered with the other samples, the post-treatment profile clustered with the rest of the non-HHT individuals (Figures 1 and 2).

## Conclusions

An individualized case-study is described for a rare disease in which we demonstrate how it is possible to progress from untargeted metabolomic profiling to identify metabolic profiles that can be then used to constrain a mechanistic network model which is then used to direct therapy decisions. Metabolomic data has been recognized as an important 'omic' data type, as it represents a quantitative biochemical phenotype and has potential to serve as a source of diagnostic and therapeutic biomarkers. As with other high-throughput data types however, a challenge with data interpretation remains. In particular, in order to achieve any practical realization of individual specific, personalized therapies, it will be important to move away from strictly statistically driven models towards more mechanistic models.

We employed the use of a global human metabolic network to interpret plasma metabolite profiling and help direct the therapy of a patient with a complex disease. Following treatment, the patient's metabolomic profile became more similar to a non-HHT individual's, suggesting the treatment was effective, at least in addressing the metabolic derangements associated with HHT. While the disease stabilized during this treatment, the patient did not undergo further cycles of treatment due to gastrointestinal intolerance of bevacizumab and long-term assessment of disease status could not be carried out. While deeper analysis with more patients are needed to further elucidate the directionality of causation, this 'individual clinical trial' illustrates how metabolomic data analyzed in the context of a network reconstruction can be potentially used to help direct therapy for complex disease states, using complicated metabolomic profiles as opposed to individual biomarkers.

One goal of personalized medicine is to analyze the data from a particular individual and determine the appropriate medication or therapeutic intervention. In this individualized case study of personalized medicine, we carry out untargeted metabolomic profiling of a patient and healthy controls. Rather than filtering the data down to a single value, these data are analyzed in the context of a network model of metabolism, in order to simulate the biochemical phenotypic differences between healthy and disease states; the results then guide the therapy. Thus, we have found that one approach to achieving the goals of individualized medicine is to use Systems Biology and causal models to drive the analysis and interpretation of data.

## Competing interests

The authors declare that they have no competing interests.

## Author's contributions

 FJM, MDK, and NJ conceived of the study. FJM, JM, TE, and MDK provided patient care and treatment management. NJ carried out the data analysis, simulations, and drafted the manuscript. NJ and MDK analyze the results. FJM, MDK, and NJ carried out extensive revisions to the manuscript. All authors read and approved the final content.

## Supplementary Material

Additional file 1 **a summary of the PCA results from the metabolomic concentration measurements**. Click here for file

Additional file 2 **An Excel spreadsheet with 5 worksheets including the metabolomic data measurements ('metabolomic_profiles'), FVA results ('simulation_FVA'), FVA summary measures ('simulation_summaries'), the reaction upper and lower bounds ('reactions'), the objective coefficients ('metabolites')**. Click here for file

Additional file 3 **an archive with the Recon 1 stoichiometric matrix (as a text file) used in the analyses**. Click here for file
