# Supplementary material for: Individualized therapy of HHT driven by network analysis of metabolomic profiles
Source: BMC Syst Biol. 2011 Dec 20;5:200. doi: 10.1186/1752-0509-5-200 (PMC3339509; doi:10.1186/1752-0509-5-200)

Principal Component Analysis was carried out on the plasma metabolite concentration data set, permitting for up to 4 different clusters. Plotting the first versus second princpal component shows three different clusters (red, green, and blue), with the largest difference separating the red versus the green and blue (i.e. pre treatment HHT versus post-treatment HHT and non-HHT individuals).

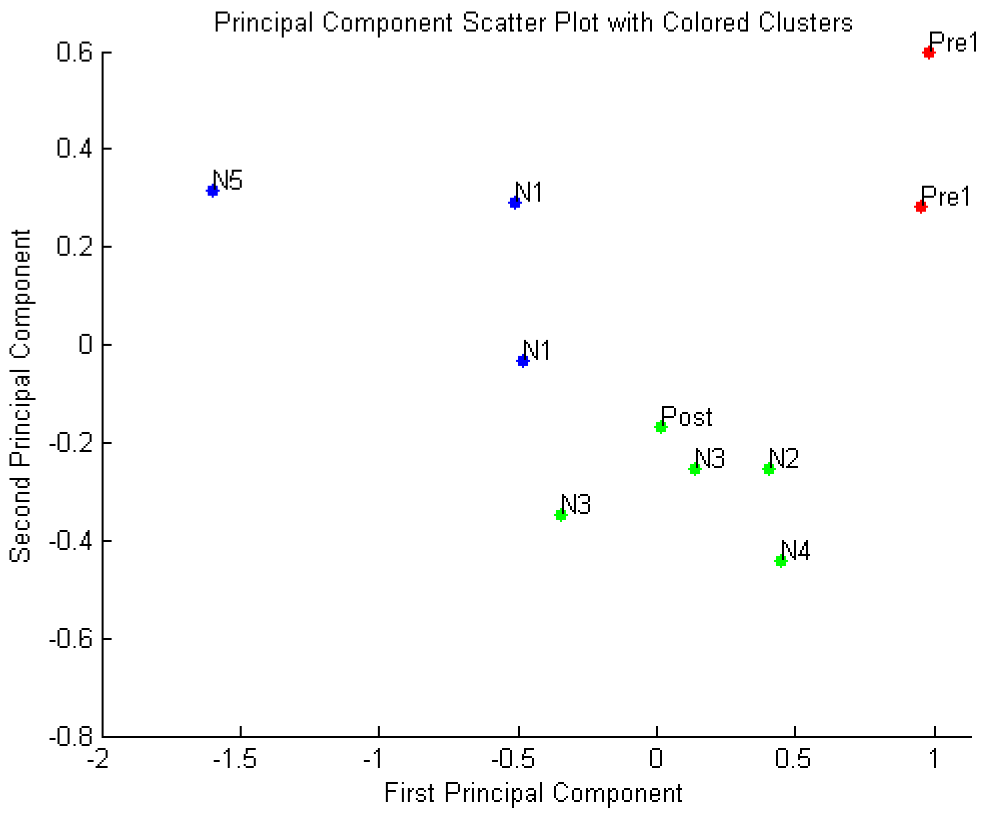

Supplement: Additional file 1 — a summary of the PCA results from the metabolomic concentration measurements. [file 1752-0509-5-200-S1.PDF]
